# Time-delay estimation in biomechanical stability: a scoping review

**DOI:** 10.3389/fnhum.2024.1329269

**Published:** 2024-01-31

**Authors:** Seyed Mohammadreza Shokouhyan, Mathias Blandeau, Laura Wallard, Franck Barbier, Kinda Khalaf

**Affiliations:** ^1^University Polytechnique Hauts-de-France, CNRS, UMR 8201 - LAMIH, Valenciennes, France; ^2^INSA Hauts-de-France, Valenciennes, France; ^3^Khalifa University of Science and Technology and Heath Innovation Engineering Center, Abu Dhabi, United Arab Emirates

**Keywords:** sensorimotor control, time delay, biomechanical model, sensory integration, balance, stability

## Abstract

Despite its high-level of robustness and versatility, the human sensorimotor control system regularly encounters and manages various noises, non-linearities, uncertainties, redundancies, and delays. These delays, which are critical to biomechanical stability, occur in various parts of the system and include sensory, signal transmission, CNS processing, as well as muscle activation delays. Despite the relevance of accurate estimation and prediction of the various time delays, the current literature reflects major discrepancy with regards to existing prediction and estimation methods. This scoping review was conducted with the aim of characterizing and categorizing various approaches for estimation of physiological time delays based on PRISMA guidelines. Five data bases (EMBASE, PubMed, Scopus, IEEE and Web of Science) were consulted between the years of 2000 and 2022, with a combination of four related categories of keywords. Scientific articles estimating at least one physiological time delay, experimentally or through simulations, were included. Eventually, 46 articles were identified and analyzed with 20 quantification and 16 qualification questions by two separate reviewers. Overall, the reviewed studies, experimental and analytical, employing both linear and non-linear models, reflected heterogeneity in the definition of time delay and demonstrated high variability in experimental protocols as well as the estimation of delay values. Most of the summarized articles were classified in the high-quality category, where multiple sound analytical approaches, including optimization, regression, Kalman filter and neural network in time domain or frequency domain were used. Importantly, more than 50% of the reviewed articles did not clearly define the nature of the estimated delays. This review presents and summarizes these issues and calls for a standardization of future scientific works for estimation of physiological time-delay.

## 1 Introduction

**FIGURE 1:**
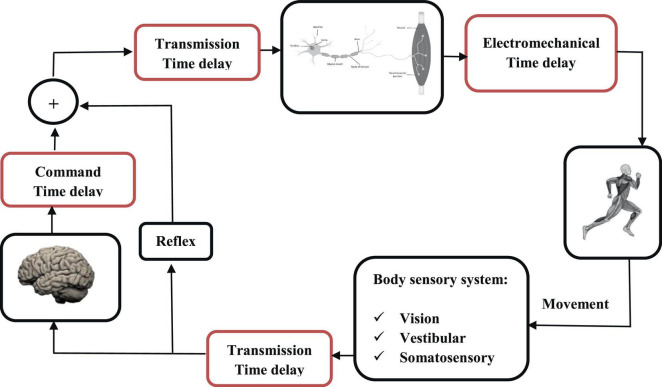
Motor control process and time-delays localization (in red).

Both biomechanical stability and balance control (treated in this review as two different biomechanical concepts on the same level) involve the central nervous system (CNS), the musculoskeletal system and the sensorimotor processes. The motor control system is considered here as the general control system in charge of the processes of initiating, directing, and grading purposeful voluntary movements in the human body. The sensorimotor control system is in charge of the internal processes within the CNS, which encompass the sensory, motor, and central integration and processing components involved in maintaining joint homeostasis during motion. Homeostasis, also referred to as functional joint stability, is defined as the dynamic process by which an organism maintains and controls its internal environment despite external perturbations (Lephart and Fu, 2000). Such system, which leverages static and dynamic components, must maintain both flexibility and functional adaptability to accommodate the notable variance among different individuals, tasks and external environmental stimuli (Riemann and Lephart, 2002). On the musculoskeletal level, the complex intersegmental dynamics of the human body (Massion, 1992), combined with the intricate muscle multiarticular structure and synergies (Latash et al., 2007; Singh et al., 2018), indicate the need for a control mechanism with a stable frame of reference, based on which postural control can be efficiently organized. This is accomplished by the CNS relying on the fusion of multiple sensory systems, such as vision, vestibular and somatosensory feedback, to maintain body stability during various daily tasks and activities. Such physiological sensory input provides valuable information for the CNS, which subsequently performs sophisticated signal integration and processing toward maintaining body stability in different postures. For example, during bipedal upright stance, the vestibular system continuously senses the angular position of the body, particularly the body’s center of mass and head displacement relative to gravity, while proprioceptive receptors sense the amplitude of force(s) from the environment (Rashid et al., 2021). The coordination of sensorimotor strategies to stabilize the body’s center of mass, during both self-initiated and externally triggered stimuli, constitutes postural equilibrium. On the other hand, these feedback signals experience various delays when transported from the sensors to the CNS. These delays, which are critical to biomechanical stability, occur throughout the sensorimotor control system, from sensory information reception to information transmission along nerve fibers, to computing responses by processing the sensory information, and feedback transmission, and to motor output in terms of muscle reaction (Figure 1).

Stabilization of an unstable condition in the presence of communication time delay is very important and yet challenging in multiple engineering applications. In biomechanical models, this is observed in dynamic systems where finding the appropriate control parameters in the presence of large time delays is not trivial for the CNS (Stépán, 1989; Gu et al., 2003). The delays generally vary depending on the distance between the sensor(s) and CNS, as well as the sensing mechanisms employed. As a consequence of conduction and neural integration time delays, reaction times are relatively long (100 s of milliseconds) and increase as the complexity of the voluntary task increases, as well as in association with aging, neurological trauma and/or disease (Gabbard, 2004).

According to the literature, the latency in motor control can be divided into three fundamental delays: delay in transmission time, electromechanical delay, and command time delay (Reeves et al., 2007; Vette et al., 2009). Transmission time corresponds to the time required for a nerve impulse to travel the path to the CNS and is a function of the transduction speed of the nerve fibers (40 and 60 m.s^–1^). The electromechanical delay, estimated at around 10 ms, corresponds to the time required for an excited muscle fiber to produce a mechanical force (Winter and Brookes, 1991; Isabelle et al., 2003). Finally, command time, which is typically the most difficult to estimate, represents the processing and response time of the CNS, in addition to the efferent transportation time. This time delay can vary significantly depending on whether the movement is voluntary (and hence processed by the brain) or reflex (without feedback to the brain). Knowing the nature of a disturbance influences the control strategy, which is then less based on a sudden reflex rather than a more modulated response (Blouin et al., 2003; Siegmund et al., 2003). The notion of attention is also relevant because cognitive load reduces the quality of postural control, particularly in the elderly, by increasing the command delay (Woollacott and Shumway-Cook, 2002). At the trunk level, many studies have measured latencies ranging from 100 to 210 ms before the production of a torque (Van Dieën et al., 1991; Thelen et al., 1994). The value of the time delay changes with health condition (Leinonen et al., 2001), age (Redfern et al., 2002), exercise (Borghuis et al., 2011), and instability (Le Mouel and Brette, 2019). Time delay plays a key role in sensorimotor control, where it is presumed to allow for optimal control over a large range of conditions in young and healthy individuals. On the other hand, difficulties to compensate for latencies are clearly observed in patients with neurological disease, such as Parkinson’s disease, MS, and Stroke, as well as among the ageing population (Leinonen et al., 2001; Redfern et al., 2002; Borghuis et al., 2011). A better understanding of the time delay can shed light on the underlying mechanisms of sensorimotor control and help design effective compensation strategies and protocols.

Up to date, time delay has been estimated based on either an experimental/clinical testing approach or by using a hybrid strategy which integrates experimental investigations with biomechanical models and simulations. Radebold et al. (2000) experimentally estimated the muscle time delay in patients with chronic low back pain (CLBP) by employing a sudden load releasing strategy in 17 chronic low back pain (CLBP) patients and 17 healthy individuals during balanced stance. The results demonstrated a significant difference in the value of muscle time delay in CLBP patients as compared with healthy individuals. In another study, Reeves et al. (2009) used a two degrees of freedom biomechanical model for seated balance, in conjunction with experimental tests, to investigate biomechanical balance in patients with CLBP as compared to healthy individuals. They hypothesized that balance during a simulated postural control task is impaired when the delay exhibited by CLBP patients is incorporated into neuromuscular control. This study employed optimization approach to minimize the error between experimental and model data to estimate the trunk muscle reflex latencies in both groups. The results reflected longer delays in the CLBP population, although instead of balance instability, both the trunk displacement and the moment increased.

In another interesting study, Mohebbi et al. (2022) used Virtual Reality (VR) stimuli in 10 healthy subjects and recorded EMG signals of four major ankle muscles during balanced standing. Using spectral analysis in the frequency domain, they estimated the total feedback time delay relative to the body’s position and produced torques, as well as the control gain of the biomechanical model. The results revealed that the gain of the body sway relative to the perturbation increased with the frequency, whereas the coherence declined. A different study (van Dieen et al., 2018) induced continuous unpredictable, force-controlled perturbations to the trunk in the anterior direction toward estimating intrinsic trunk stiffness and damping, as well as feedback gains and delays in muscle spindles, Golgi tendon organs and the vestibular system. Frequency response functions (FRFs) of the amount of movement per unit force were obtained, and several physiological models were fitted based on the FRFs. The authors concluded that muscle spindle feedback and intrinsic mechanical properties were sufficient to describe trunk stabilization in the sagittal plane subject to small mechanical perturbations.

Due to the various experimental protocols used to estimate the time delays inherent to the sensorimotor control system, the literature is riddled with different time delay values as associated with physiological response time (e.g., muscle activation response time or total sensorimotor control time delay, etc.). Therefore, the aim of this review is to summarize and analyze the results of multiple relevant clinical and modeling/simulation studies. Such review can provide context for the current heterogeneity of these parameters in literature toward standardization and better understanding of the underlying mechanisms of human sensory motor control. The remainder of this paper is organized as follows: section “2 Methods” describes the methodology, including the PRISMA search strategy, data extraction and analyses, quality assessment, and data collection and analysis. Section “3 Results” presents the results, including a summary of the identified articles, types of estimated time delay, experimental protocols, perturbations, computational approaches, and simulation models. The results are discussed in Section “4 Discussion,” focusing on the definition of time delay in the context of various experimental protocols and analytical approaches, followed by a brief discussion of the limitations of the review. Conclusive remarks are presented in Section “5 Limitations.”

## 2 Methods

### 2.1 Search strategy

This scoping review adhered to the Preferred Reporting for Systematic review and Meta-Analysis (PRISMA) guidelines (Tricco et al., 2018). Electronic literature databases, including EMBASE, PubMed, Scopus, IEEE and the Web of Science were searched for 22 years of relevant publications (between January 2000 and June 2022). Four groups of keywords related to time delay, biomechanical modeling of body, sensory integration, and postural control were utilized. Related keywords covering all MeSH terms were used in a comprehensive way by using “AND” and “OR” Boolean operators in order to combine all the keywords in each group as well as all groups, where the combinations can be seen in Table 1.

**TABLE 1:** Groups of keywords and the combination used in this review.

A	(“Time delay” OR “time-delay” OR “time delays” OR “time-delays”)
B	(“Neural controller” OR “proprioceptive feedback” OR “vestibular feedback” OR “vision feedback” OR “feedback control” OR “human balance” OR “neuromuscular feedback” OR “postural control” OR “postural balance” OR “postural stability” OR “postural sway” OR “postural instability”)
C	(“Biomechanical model*” OR “human feedback model*” OR “human balance model*” OR “human postural control model*” OR “neuromuscular model*” OR “mathematical model*” OR “mechanical model*” OR “human body model*” OR “Musculoskeletal model*” OR “skeletal model*”)
D	(“Sensory integration” OR “Sensorimotor integration”)
Algorithm	{A AND [B OR C OR D]} OR {B AND [C OR D OR A]} OR {C AND [D OR A OR B]}OR {D AND [A OR B OR C]}

### 2.2 Data extraction and analyses

All of articles identified through the search strategy in the various databases, carried out by the first author (SMS), were imported to Zotero, where any duplications were removed using the Zotero software. Titles and abstracts were then reviewed by two 2 independent members (SS and MB) of the research team based on the following inclusion criteria: (1) the study published after 2000, (2) full scientific paper, (3) the study estimated one of physiological time delays by experimental set up and/or via biomechanical modeling/simulation, and (4) the study was written in English. For each study that met the inclusion criteria, the full text was retrieved, analyzed, and evaluated by the same two authors. Any conflict was resolved by discussion. Due to methodological heterogeneity among the studies, there was a lack of comparative data, and a meta-analysis could not be performed; therefore, the data are presented descriptively.

### 2.3 Quality assessment

Two authors (LW and FB) identified and extracted 16 appropriate qualification questions from the studies (Peters et al., 2010; Lempereur et al., 2014; Desmyttere et al., 2018) in order to assess the quality of writing and organization of each article. Values from 0 to 2 were used to score the article in each of the qualification questions, where the value “0” represented “no description,” “1” indicated “limited description” and the value of “2” referred to “full description.” Studies with the quality score of 75% or higher were classified as high quality, those with 60–74% as moderate quality, and those 60% or less as low quality (Radzimski et al., 2012; Hajizadeh et al., 2016). Disagreement in the scoring responses after the review process was discussed by all authors. In addition to the qualitative assessment, 20 additional quantification questions were created by the reviewers to assess the physiological and sensorimotor concepts regarding the time delay estimation. Both qualification and quantification questions are depicted in the Table 2.

**TABLE 2:** Quantification and qualification questions used in the review.

Quantification questions	Qualification questions
1. Which postural balance was analyzed?	1. Is the study design clearly described in the abstract?
2. Did the study perform any Experimental tests?	2. Are the research objectives clearly stated?
3. Did the study perform any theoretical simulation?	3. Were participant characteristics adequately described?
4. Was the model totally linearized?	4. Are the estimation method principles clearly explained?
5. Did the study use linearization in some part of the model?	5. Are implementation details provided?
6. Was the sensory system modeled?	6. Were movement tasks clearly defined?
7. Was the muscular system modeled?	7. Was equipment design and set up clearly described?
8. What was the value of time delay used in the model?	8. Were the evaluation strategy and the reference used appropriately justified?
9. Was the time delay the only parameter estimated?	9. Were the analytical techniques clearly described?
10. Was the Time delay constant or variable?	10. Was the time delay estimated on repeated measurement?
11. What estimation tool was used in the study? (e.g., Matlab)	11. Were the statistical methods justified and appropriately described?
12. What estimation method was used in the study for time delay estimation?	12. Were direct results easily interpretable?
13. What clinical measurement was used for the estimation? (EMG data or …)	13. Were the main outcomes clearly stated and supported by the results?
14. Were the subjects healthy or patient?	14. Were limitations of the study clearly described?
15. What kind of perturbation was used? (External or Internal)	15. Were key findings positioned with respect to the state-of-the-art?
16. In which direction the perturbation was exerted? (AP or ML)	16. Were conclusions drawn from the study clearly stated?
17. Was the perturbation “expected” or “unexpected”?	
18. Was the amplitude of perturbation normalized?	
19. Did the participants use pre-activated muscle or pre-information of their sensory systems?	
20. Was the nature of the time delay described? and which time delay was estimated? (CNS, sensory, Muscle)	

### 2.4 Data collection process and analysis

The included articles were categorized into several subgroups based on the nature of the time delay, using experimental data or a combined approach with both modeling/simulation and experimental data. Thus, the quantification questions, shown in Table 2, were designed to evaluate the articles technically. Effective variables in the time delay estimation were assessed by these questions.

## 3 Results

### 3.1 Identified articles

**FIGURE 2:**
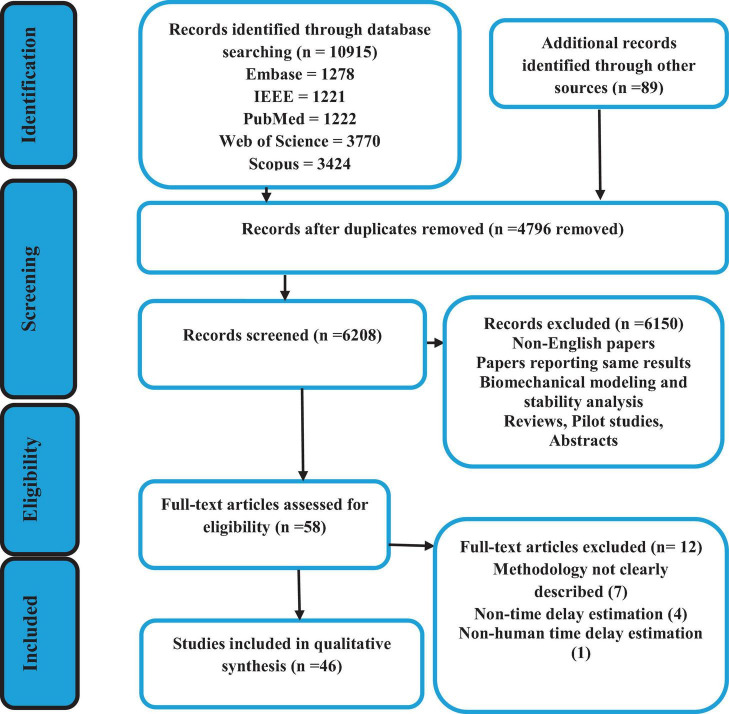
Flow diagram of the search selection process.

Five databases identified 10,915 articles using the inclusion criteria discussed in section “2.2 Data extraction and analyses” and the combination of the search terminology shown in Figure 2. The details of these articles, as well as the search selection process are also depicted in Figure 2. Out of the total number of articles, 4,795 were excluded due to duplication. Therefore, the titles and abstracts of 6,209 articles were screened, and based on the inclusion criteria, 58 articles were found eligible for full-text screening (Figure 2). Eventually, 46 articles were identified to be analyzed based on the previously described quantification and qualification questions.

### 3.2 Quality assessment results

**FIGURE 3:**
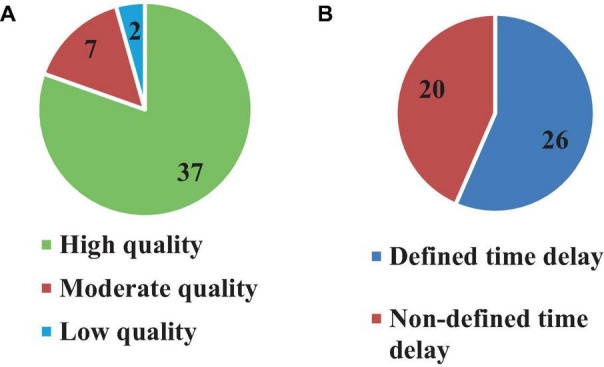
Number of articles based on quality **(A)** and time delay definition **(B)**.

All 46 articles were evaluated based on 16 qualification questions (Table 3), yielding a Mean ± SD value of 79.7 ± 11.7 for the qualification analysis. Most of the included articles (Radebold et al., 2000, 2001; Brown et al., 2001; Leinonen et al., 2001; Adkin et al., 2002; Redfern et al., 2002; Dimitrova et al., 2004; Granata et al., 2004; Peterka and Loughlin, 2004; Cholewicki et al., 2005; Reeves et al., 2005, 2018; Masani et al., 2008; Mochizuki et al., 2008; Vette et al., 2008; Welch and Ting, 2008, 2009; Loram et al., 2009; Santos et al., 2010; Borghuis et al., 2011; Mohapatra et al., 2012; Weaver et al., 2012; Liebetrau et al., 2013; van Drunen et al., 2013; Kanekar and Aruin, 2014a,b; Pereira et al., 2014; Valles et al., 2014; Aruin et al., 2015; Blenkinsop et al., 2016; Fujio et al., 2016; Claudino et al., 2017; van Dieen et al., 2018; Curuk et al., 2020; Molnar et al., 2021; Wang and van den Bogert, 2021; Mohebbi et al., 2022) were classified as high quality (37, 80.4%) in terms of research objectives, subjects’ characteristics, experimental protocol, data analysis and conclusion. However, the limitation of these studies was not always discussed. In addition, 7 (15.2%) articles (Peterka, 2002; Maurer and Peterka, 2005; Reeves et al., 2009; Murai et al., 2010; Nagy et al., 2020; Yin et al., 2020; Zelei et al., 2021) were found at the moderate quality level, and finally, 2 (4.4%) articles scored as low quality (Sovol et al., 2010; McKee and Neale, 2019). Detail characteristic information for all included articles is shown in Table 3 and Figure 3.

**TABLE 3:** Quantification and qualification analysis results of the included studies.

Referen-ces	Quantification questions																			
	1	2	3	4	5	6	7	8	9	10	11	12	13	14	15	16	17	18	19	20
Leinonen et al., 2001	Seated balance	Yes	No	nd	nd	nd	nd	30–60	Yes	Constant	MATLAB	Direct analysis of clinical data (MAOD**)	EMG and kinematics	Healthy and CLBP	External	AP	Expected and unexpected	No	Yes	No
Redfern et al., 2002	Stance balance	Yes	No	nd	nd	nd	nd	150–300	Yes	Constant	MATLAB	Direct analysis of clinical data (first maximum of the second derivative of the cop signal)	COP	Healthy	External	AP	Unexpected	No	No	Yes, total time delay
Borghuis et al., 2011	Seated balance	Yes	No	nd	nd	nd	nd	80–110	Yes	Constant	MATLAB	Direct analysis of clinical data (Variance ratio)	EMG and trunk kinematic	Healthy	External	AP and ML	Unexpected	No	No	No
Radebold et al., 2000	Seated balance	Yes	No	nd	nd	nd	nd	70–83	Yes	Constant	MATLAB	Direct analysis of clinical data (MAOD**)	EMG	Healthy and LBP	External	AP and ML	Unexpected	Yes	No	Yes, total time delay
Reeves et al., 2009	Seated balance	Yes	Yes	Yes	Yes	Yes	No	13–32	Yes	Constant	MATLAB	Optimization	Trunk kinematics	Healthy	External	AP	Unexpected	No	Yes	Yes, total delay
Mohebbi et al., 2022	Stance balance	Yes	No	nd	nd	nd	nd	163–195	No	Constant	MATLAB	Direct analysis of clinical data (sign change of signal’s derivative)	EMG, Angle, Ankle Torque	Healthy	Internal	nd	Unexpected	No	No	No
van Dieen et al., 2018	Seated balance	Yes	Yes	Yes	Yes	Yes	Yes	5–200	No	Constant	MATLAB	Simulation and data fitting	EMG and, Kinetic and Kinematic data	Healthy	External	AP	Unexpected	No	No	No
Reeves et al., 2018	Seated balance	Yes	No	nd	nd	nd	nd	350–550	Yes	Constant	MATLAB	Direct analysis of clinical data (applying a delayed transfer function)	Trunk kinematics	Healthy	External	AP	Expected and unexpected	No	No	Yes, total time delay
Peterka and Loughlin, 2004	Stance balance	Yes	Yes	Yes	Yes	Yes	No	175	No	Constant	MATLAB	Frequency Analysis (energy ratio in time-frequency distribution)	COP	Healthy	External	AP	Unexpected	No	No	Yes, total time delay
Masani et al., 2008	Stance balance	Yes	Yes	Yes	Yes	Yes	No	121–192	No	Constant	MATLAB	Optimization	EMG	Healthy	No perturba-tion	nd*	nd	nd	No	Yes, neural trans-mission
Blenkinsop et al., 2016	Hand stand	Yes	No	nd	nd	nd	nd	142–220	No	Constant	MATLAB	Direct analysis of clinical data (cross correlation and delayed regression models)	EMG, Kinematic data and COP	Healthy	External	AP	Unexpected	No	No	Yes, total delay
Molnar et al., 2021	Stance balance	Yes	Yes	Yes	Yes	Yes	No	169–314	Yes	Constant	MATLAB	Simulation and experimental tests	Kinematic data	Healthy	No perturba-tion	nd	nd	nd	Yes	Yes, total time delay
Claudino et al., 2017	Stance balance	Yes	No	nd	nd	nd	nd	50–133	Yes	Constant	MATLAB	Direct analysis of clinical data (onset detection in EMG, COP signals)	EMG and COP	Healthy	External	ML	Unexpected	Yes	Yes	No
Liebetrau et al., 2013	Stance balance	Yes	Yes	No	No	Yes	Yes	38–85	Yes	Constant	MATLAB	Direct analysis of clinical data (MAOD**)	EMG	Healthy and CLBP	External	ML	Unexpected	No	No	No
Mohapatra et al., 2012	Stance balance	Yes	No	nd	nd	nd	nd	40–140	Yes	Constant	MATLAB	Direct analysis of clinical data (MAOD**)	EMG and COP	Healthy	External	AP	Expected and unexpected	No	Yes	No
Loram et al., 2009	Balancing a virtual inverted pendulum	Yes	No	nd	nd	nd	nd	114–250	Yes	Constant	MATLAB	Direct analysis of clinical data (closed loop impulse response and cross correlation functions)	Kinematic data	Healthy	Internal, voltage white noise	nd	Unexpected	No	No	No
Radebold et al., 2001	Seated balance	Yes	No	nd	nd	nd	nd	48–80	Yes	Constant	MATLAB	Direct analysis of clinical data (MAOD**)	EMG and seatting COP	Healthy and CLBP	External	AP and ML	Unexpected	Yes	Yes	Yes, total time delay
Weaver et al., 2012	Stance balance	Yes	No	nd	nd	nd	nd	147–217	Yes	Constant	Spike 2, Cambridge Electronic Design, Cambridge, United Kingdom	Direct analysis of clinical data (MAOD**)	EMG	Healthy	External	AP	Expected and unexpected	No	Yes	No
Granata et al., 2004	Seated balance	Yes	No	nd	nd	nd	nd	25–50	Yes	Constant	MATLAB	Direct analysis of clinical data (MAOD**) and regression	EMG	Healthy	External	AP	Unexpected	No	Yes	No
Cholewicki et al., 2005	Seated balance	Yes	No	nd	nd	nd	nd	48–77	Yes	Constant	MATLAB	Direct analysis of clinical data and regression (MAOD**)	EMG	Healthy and LBP	External	AP and ML	Unexpected	Yes	Yes	No
Welch and Ting, 2009	Stance balance	Yes	Yes	Yes	Yes	Yes	Yes	60–116	No	Constant	MATLAB	Simulation and optimization with clinical data	EMG, Kinematic data and GRF	Healthy	External	AP	Unexpected	No	No	No
Reeves et al., 2005	Seated balance	Yes	No	nd	nd	nd	nd	62–99	Yes	Constant	MATLAB	Direct analysis of clinical data and regression (MAOD**)	EMG	Healthy and LBP	External	AP and ML	Unexpected	Yes	Yes	No
van Drunen et al., 2013	Seated balance	Yes	Yes	No	Yes	Yes	Yes	15–50	No	Constant	MATLAB	Simulation and optimization and data fitting	EMG and kinematic data	Healthy	External	AP	Unexpected	No	No	No
Wang and van den Bogert, 2021	Stance balance	Yes	Yes	No	No	Yes	No	50–120	No	Constant	MATLAB	Simulation and optimization with clinical data	Body angles	Healthy	External	AP	Unexpected	No	No	No
Curuk et al., 2020	Stance balance	Yes	No	nd	nd	nd	nd	20–150	Yes	Constant	MATLAB	Direct analysis of clinical data (MAOD**)	EMG	Healthy and Stroke patients	External	AP	Expected	Yes	Yes	Yes, total delay
Welch and Ting, 2008	Stance balance	Yes	Yes	Yes	Yes	Yes	Yes	20–80	No	Constant	MATLAB	Simulation and optimization with clinical data	EMG and body kinematics	Healthy	External	AP	Unexpected	No	No	Yes, total delay
Vette et al., 2008	Stance balance	Yes	Yes	Yes	Yes	Yes	Yes	253	No	Constant	MATLAB	Simulation and optimization and data fitting	EMG and body kinematics and Ankle torque	Healthy	No perturba-tion	nd	nd	Nd	No	Yes, twitch contrac-tion time
Valles et al., 2014	Stance balance	Yes	Yes	Yes	Yes	Yes	No	110–131	No	Constant	MATLAB	Simulation and optimization and data fitting	COP	Healthy	No perturba-tion	nd	nd	nd	No	Yes, trans-mission and process-ing delay
Fujio et al., 2016	Stance balance and supine position	Yes	No	nd	nd	nd	nd	43–93	Yes	Constant	MATLAB	Direct analysis of clinical data (MAOD**)	EMG	Healthy	External	AP	Expected and unexpected	No	Yes	Yes, total delay
Mochizuki et al., 2008	Stance balance	Yes	No	nd	nd	nd	nd	130–150	No	Constant	MATLAB	Direct analysis of clinical data (onset detection in EMG, COP and EEG signals)	EMG, COP and EEG	Healthy	External	AP	Expected and unexpected	No	No	No
Pereira et al., 2014	Stance balance	Yes	No	nd	nd	nd	nd	50	Yes	Constant	MATLAB	Direct analysis of clinical data (MAOD**)	EMG	Healthy and Stroke patients	No perturba-tion	nd	nd	nd	No	Yes, total delay (APA)
Aruin et al., 2015	Stance balance	Yes	No	nd	nd	nd	nd	50–380	Yes	Constant	MATLAB	Direct analysis of clinical data (MAOD**)	EMG	Old Healthy	External	AP	Expected and unexpected	Yes	Yes	Yes, total delay (APA)
Santos et al., 2010	Stance balance	Yes	No	nd	nd	nd	nd	70–200	Yes	Constant	MATLAB	Direct analysis of clinical data (MAOD**)	EMG	Healthy	External	AP	Expected and unexpected	Yes	Both	Yes, total delay (APA)
Kanekar and Aruin, 2014a	Stance balance	Yes	No	nd	nd	nd	nd	150–300	Yes	Constant	MATLAB	Direct analysis of clinical data (MAOD**)	EMG	Healthy (young and old)	External	AP	Expected	Yes	Yes	Yes, total delay (APA)
Kanekar and Aruin, 2014b	Stance balance	Yes	No	nd	nd	nd	nd	50–100	Yes	Constant	MATLAB	Direct analysis of clinical data (MAOD**)	EMG	Healthy (old)	External	AP	Expected and unexpected	Yes	Both	Yes, total delay (APA)
Dimitrova et al., 2004	Stance balance	Yes	No	nd	nd	nd	nd	150	Yes	Constant	MATLAB	Direct analysis of clinical data (MAOD**)	EMG	Healthy and PD patients	External	AP and ML	Unexpected	No	nd	Yes, total delay (APA)
Brown et al., 2001	Stance balance	Yes	No	nd	nd	nd	nd	100	Yes	Constant	MATLAB	Direct analysis of clinical data (onset detection in EMG, COM and ankle torque signals)	EMG, COM and ankle torque	Healthy	External	AP	Expected	No	Yes	Yes, total delay (APA)
Adkin et al., 2002	Stance balance	Yes	No	nd	nd	nd	nd	250	Yes	Constant	MATLAB	Direct analysis of clinical data (onset detection in EMG, COP signals)	EMG and COP	Healthy	No perturba-tion	nd	nd	nd	Yes	Yes, total delay (APA)
Murai et al., 2010	Stepping, jump and squat	Yes	Yes	No	No	Yes	Yes	25–150	Yes	Constant	MATLAB	Optimization and neural network	EMG and Kinematic data	Healthy	No perturba-tion	nd	nd	nd	No	Yes, neural trans-mission
Nagy et al., 2020	Stick balancing	Yes	Yes	Yes	Yes	Yes	No	90–450	Yes	Constant	MATLAB	Experiment and Cepstral analysis	Kinematic data	Healthy	No perturba-tion	nd	nd	nd	No	No
Peterka, 2002	Stance balance	Yes	Yes	Yes	Yes	Yes	No	206 and 191	No	Constant	MATLAB	Simulation and data fitting	COP and kinematic data	Healthy and vestibular loss	External	AP	Unexpected	No	No	No
Zelei et al., 2021	Stance balance	Yes	Yes	Yes	Yes	Yes	No	104–211	No	Constant	MATLAB	Simulation and data fitting	Kinematic data	Healthy	External	AP	Unexpected	Yes	Yes	Yes, total time delay
Yin et al., 2020	Stance balance	Yes	Yes	No	Yes	Yes	Yes	120–180 and 45–70	No	Constant	MATLAB	Simulation and optimization with clinical data	EMG, body Angles	Healthy	External	AP	Unexpected	No	No	Yes, total time delay, neural trans-mission and process-ing delay
Maurer and Peterka, 2005	Stance balance	Yes	Yes	No	No	Yes	No	165	No	Constant	MATLAB	Simulation and optimization and data fitting	COP	Healthy	No perturba-tion	nd	nd	nd	No	Yes, neural trans-mission and process-ing delay
McKee and Neale, 2019	Stance balance	Yes	Yes	No	Yes	Yes	No	200	No	Constant	MATLAB	Simulation and Kalman filter with clinical data	COM	Healthy	No perturba-tion	nd	nd	nd	No	No
Sovol et al., 2010	Stance balance	Yes	Yes	Yes	Yes	Yes	No	170	No	Constant	MATLAB	Optimization and data fitting	COP	Children with diplegic cerebral palsy	No perturba-tion	nd	nd	nd	nd	No
**Qualification questions**																				
	**1**		**2**	**3**	**4**	**5**	**6**	**7**	**8**	**9**		**10**	**11**	**12**	**13**	**14**	**15**	**16**	**Per%**	
Leinonen et al., 2001	2		2	2	2	1	2	2	2	2		2	2	2	2	1	2	2	93.8	
Redfern et al., 2002	1		2	1	2	1	2	2	2	2		2	2	2	1	0	2	2	81.3	
Borghuis et al., 2011	2		2	2	1	1	2	2	2	1		2	2	1	1	0	2	2	78.1	
Radebold et al., 2000	2		2	2	2	2	2	2	2	2		2	2	2	2	0	2	2	93.8	
Reeves et al., 2009	2		2	2	2	1	2	2	2	1		2	0	0	1	0	2	2	71.9	
Mohebbi et al., 2022	2		2	2	2	1	2	2	2	1		2	2	2	2	0	2	2	87.5	
van Dieen et al., 2018	2		2	1	2	1	2	2	2	1		2	0	2	2	2	1	2	81.3	
Reeves et al., 2018	2		2	1	2	1	2	2	2	1		2	1	2	2	2	2	2	87.5	
Peterka and Loughlin, 2004	2		2	1	1	2	2	2	2	2			1	2	1	0	2	2	81.3	
Masani et al., 2008	2		2	1	2	1	2	2	2	2		0	1	2	2	2	1	2	81.3	
Blenkinsop et al., 2016	2		2	2	2	1	2	2	2	1		2	2	2	2	0	2	2	87.5	
Molnar et al., 2021	2		2	2	2	1	2	2	2	2		0	0	2	2	0	2	2	78.1	
Claudino et al., 2017	2		2	2	2	0	2	2	2	0		2	2	2	2	2	2	2	87.5	
Liebetrau et al., 2013	2		2	2	2	1	2	2	2	2		2	2	2	2	2	2	2	96.9	
Mohapatra et al., 2012	1		2	1	2	1	2	2	1	1		2	2	2	2	2	2	2	84.4	
Loram et al., 2009	2		2	1	2	1	2	2	2	1		0	2	1	2	0	2	2	75	
Radebold et al., 2001	2		2	2	2	1	2	2	2	1		2	2	2	2	0	2	2	87.5	
Weaver et al., 2012	2		2	2	2	1	2	2	2	1		0	2	2	1	0	2	2	78.1	
Granata et al., 2004	2		2	1	2	1	2	2	2	1		0	1	2	2	0	2	2	75	
Cholewicki et al., 2005	2		2	1	1	0	2	2	2	1		2	2	2	2	2	2	2	84.4	
Welch and Ting, 2009	2		2	1	1	1	2	2	2	1		2	2	2	2	0	2	2	81.3	
Reeves et al., 2005	2		2	2	1	0	2	2	2	1		2	2	2	2	2	2	2	87.5	
van Drunen et al., 2013	2		2	1	2	2	2	2	2	2		2	1	2	2	0	2	2	87.5	
Wang and van den Bogert, 2021	2		2	2	2	1	2	2	2	1		0	0	2	2	2	1	2	78.1	
Curuk et al., 2020	2		2	2	2	2	2	2	2	2		2	2	1	2	2	2	2	96.9	
Welch and Ting, 2008	2		2	1	2	1	2	2	2	1		2	0	2	2	0	2	1	75	
Vette et al., 2008	2		2	1	2	1	2	2	2	1		0	0	2	2	2	1	2	75	
Valles et al., 2014	2		2	2	2	1	2	2	2	1		0	2	1	2	0	2	2	78.1	
Fujio et al., 2016	2		2	2	2	2	2	2	2	1		0	2	2	2	2	2	2	90.6	
Mochizuki et al., 2008	2		2	2	2	2	2	2	2	1		0	2	1	2	0	1	2	78.1	
Pereira et al., 2014	2		2	2	2	2	2	2	2	1		2	2	2	2	2	2	2	96.9	
Aruin et al., 2015	2		2	2	2	2	2	2	2	1		2	1	2	2	0	2	2	87.5	
Santos et al., 2010	2		2	1	2	2	2	2	2	1		2	1	2	2	0	2	2	84.4	
Kanekar and Aruin, 2014a	2		2	1	2	2	2	2	2	2		2	2	2	2	0	2	2	90.6	
Kanekar and Aruin, 2014b	2		1	1	2	2	2	2	2	1		2	2	2	2	0	2	2	84.4	
Dimitrova et al., 2004	2		2	2	2	1	2	1	2	1		0	2	2	2	0	2	2	78.1	
Brown et al., 2001	1		2	1	2	1	2	2	2	1		2	2	2	2	0	2	2	81.3	
Adkin et al., 2002	2		2	2	1	2	2	2	2	1		2	2	2	2	0	2	2	87.5	
Murai et al., 2010	2		2	1	2	0	2	2	2	0		0	0	2	2	0	2	2	65.6	
Nagy et al., 2020	2		1	0	2	0	2	2	2	1		1	0	2	2	0	2	2	65.6	
Peterka, 2002	2		1	2	2	1	2	2	2	1		0	1	1	1	0	2	2	68.8	
Zelei et al., 2021	2		2	2	1	0	2	2	2	2		0	0	2	2	0	2	1	68.8	
Yin et al., 2020	1		2	1	1	2	2	2	2	1		0	0	2	2	0	2	2	68.8	
Maurer and Peterka, 2005	2		2	0	2	1	0	0	2	1		0	2	2	2	0	2	2	62.5	
McKee and Neale, 2019	2		2	0	2	2	1	0	2	2		0	0	0	2	0	2	2	59.4	
Sovol et al., 2010	0		1	0	1	0	0	0	1	1		0	0	2	1	0	2	1	31.3	

nd*No description was provided.

MAOD**Muscle activation onset detection.

### 3.3 Types of estimated time delay

**FIGURE 4:**
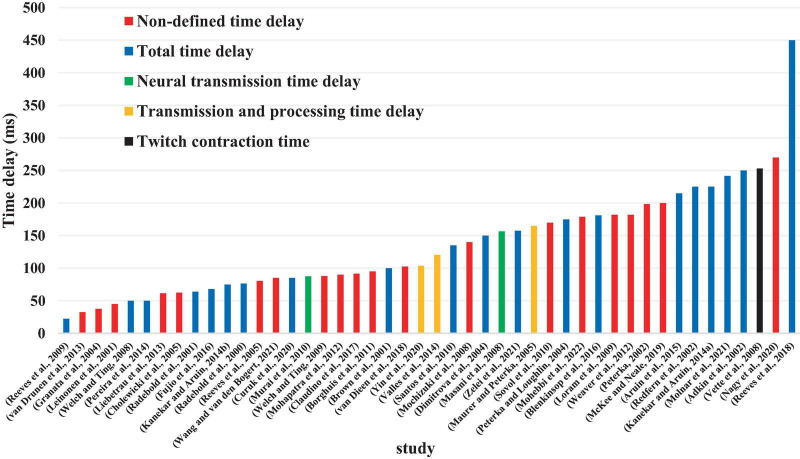
Value of estimated time delay in each study.

**FIGURE 5:**
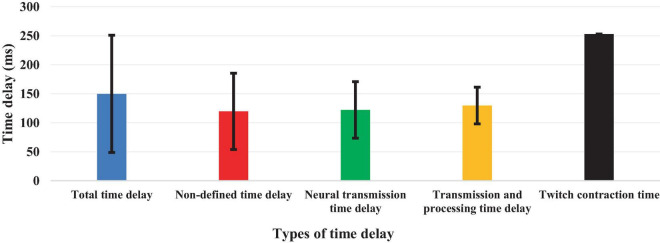
Mean and standard deviation values of each type of estimated time delay.

Although 26 articles (56.5%) specified (Radebold et al., 2000, 2001; Brown et al., 2001; Adkin et al., 2002; Redfern et al., 2002; Dimitrova et al., 2004; Peterka and Loughlin, 2004; Maurer and Peterka, 2005; Masani et al., 2008; Vette et al., 2008; Welch and Ting, 2008; Reeves et al., 2009, 2018; Murai et al., 2010; Santos et al., 2010; Kanekar and Aruin, 2014a,b; Pereira et al., 2014; Valles et al., 2014; Aruin et al., 2015; Blenkinsop et al., 2016; Fujio et al., 2016; Curuk et al., 2020; Yin et al., 2020; Molnar et al., 2021; Zelei et al., 2021) the type of estimated time delay (Figure 3), 20 articles (43.5%) did not mention it (Leinonen et al., 2001; Peterka, 2002; Granata et al., 2004; Cholewicki et al., 2005; Reeves et al., 2005; Mochizuki et al., 2008; Loram et al., 2009; Welch and Ting, 2009; Sovol et al., 2010; Borghuis et al., 2011; Mohapatra et al., 2012; Weaver et al., 2012; Liebetrau et al., 2013; van Drunen et al., 2013; Claudino et al., 2017; van Dieen et al., 2018; McKee and Neale, 2019; Nagy et al., 2020; Wang and van den Bogert, 2021; Mohebbi et al., 2022). Moreover, different values of time delay were reported in these studies, where notably most of the articles estimated the total time delay (the delay consisting of sensory detection, CNS processing, signal transmission and muscle activation). Some articles provided estimates of the time delay based on muscle activation and anticipatory and compensatory postural adjustments (Brown et al., 2001; Adkin et al., 2002; Dimitrova et al., 2004; Masani et al., 2008; Vette et al., 2008; Welch and Ting, 2008; Santos et al., 2010; Kanekar and Aruin, 2014b,a; Pereira et al., 2014; Aruin et al., 2015; Fujio et al., 2016; Curuk et al., 2020). Figure 4 presents the estimated delay values of all articles, where the average delay amounted to 150 ms. In addition, the mean and standard deviation values of each type of time delay are depicted in Figure 5.

### 3.4 Experimental protocols and subjects

**FIGURE 6:**
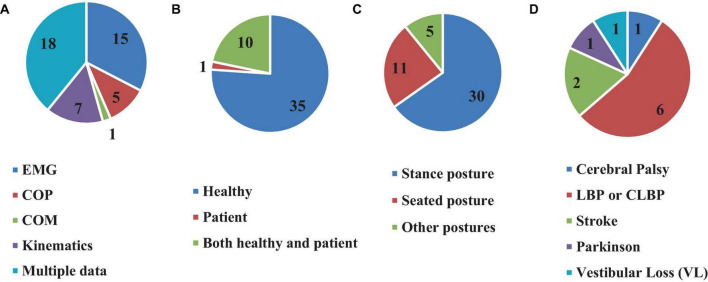
Number of articles based on using different clinical data **(A)**, participated subjects **(B)**, types of posture **(C)**, and different patients **(D)** for time delay estimation.

All 46 reviewed articles used experimental investigations for estimating the time delay, among which 35 articles (76%) used only healthy individuals (Brown et al., 2001; Adkin et al., 2002; Redfern et al., 2002; Granata et al., 2004; Peterka and Loughlin, 2004; Maurer and Peterka, 2005; Masani et al., 2008; Mochizuki et al., 2008; Vette et al., 2008; Welch and Ting, 2008, 2009; Loram et al., 2009; Murai et al., 2010; Santos et al., 2010; Borghuis et al., 2011; Mohapatra et al., 2012; Weaver et al., 2012; van Drunen et al., 2013; Kanekar and Aruin, 2014a,b; Valles et al., 2014; Aruin et al., 2015; Blenkinsop et al., 2016; Fujio et al., 2016; Claudino et al., 2017; Reeves et al., 2018; van Dieen et al., 2018; McKee and Neale, 2019; Nagy et al., 2020; Yin et al., 2020; Molnar et al., 2021; Wang and van den Bogert, 2021; Zelei et al., 2021; Mohebbi et al., 2022), 1 article (2.1%) tested Cerebral Palsy (CP) patients (Sovol et al., 2010), while 10 articles (21.7%) investigated both healthy subjects and patients (Radebold et al., 2000, 2001; Leinonen et al., 2001; Peterka, 2002; Dimitrova et al., 2004; Cholewicki et al., 2005; Reeves et al., 2005; Liebetrau et al., 2013; Pereira et al., 2014; Curuk et al., 2020) in which these studies can be classified into five groups of patients in terms of pathology or disease. These include LBP or CLBP: (six studies) (Radebold et al., 2000, 2001; Leinonen et al., 2001; Cholewicki et al., 2005; Reeves et al., 2005; Liebetrau et al., 2013); CP: (one study) (Sovol et al., 2010); stroke: (two studies) (Pereira et al., 2014; Curuk et al., 2020); Parkinson’s Disease : (one study) (Dimitrova et al., 2004); and vestibular loss : (one study) (Peterka, 2002; Figure 6). Moreover, several types of physiological signals were included in the time delay estimation. Among the 28 articles which analyzed only one signal, electromyography (EMG) depicting muscle activity was the most used (15 articles, 32.6%) (Radebold et al., 2000; Dimitrova et al., 2004; Granata et al., 2004; Cholewicki et al., 2005; Reeves et al., 2005; Masani et al., 2008; Santos et al., 2010; Weaver et al., 2012; Liebetrau et al., 2013; Kanekar and Aruin, 2014a,b; Pereira et al., 2014; Aruin et al., 2015; Fujio et al., 2016; Curuk et al., 2020). This was followed by segmental and intersegmental joint kinematics (joint angular position or segment motion) [Kinematics, 7 articles (15.21%)] (Loram et al., 2009; Reeves et al., 2009, 2018; Nagy et al., 2020; Molnar et al., 2021; Wang and van den Bogert, 2021; Zelei et al., 2021), center of pressure (COP, 5 articles, 10.8%) (Redfern et al., 2002; Peterka and Loughlin, 2004; Maurer and Peterka, 2005; Sovol et al., 2010; Valles et al., 2014), and center of mass (COM, 1 article, 2.1%) (McKee and Neale, 2019). On the other hand, 18 articles (39.1%) articles used multimodal physiological data (EMG, COP and body kinematics) simultaneously for time delay estimation (Brown et al., 2001; Leinonen et al., 2001; Radebold et al., 2001; Adkin et al., 2002; Peterka, 2002; Mochizuki et al., 2008; Vette et al., 2008; Welch and Ting, 2008, 2009; Murai et al., 2010; Borghuis et al., 2011; Mohapatra et al., 2012; van Drunen et al., 2013; Blenkinsop et al., 2016; Claudino et al., 2017; van Dieen et al., 2018; Yin et al., 2020; Mohebbi et al., 2022; Figure 6).

### 3.5 Postures and stabilizing tasks

Furthermore, the value of the estimated time delay may vary due to investigating different postures. For instance, among the articles reviewed here, 11 assessed seating posture (Radebold et al., 2000, 2001; Leinonen et al., 2001; Granata et al., 2004; Cholewicki et al., 2005; Reeves et al., 2005, 2009, 2018; Borghuis et al., 2011; van Drunen et al., 2013; van Dieen et al., 2018), 30 investigated stance posture (Brown et al., 2001; Adkin et al., 2002; Peterka, 2002; Redfern et al., 2002; Dimitrova et al., 2004; Peterka and Loughlin, 2004; Maurer and Peterka, 2005; Masani et al., 2008; Mochizuki et al., 2008; Vette et al., 2008; Welch and Ting, 2008, 2009; Santos et al., 2010; Sovol et al., 2010; Mohapatra et al., 2012; Weaver et al., 2012; Liebetrau et al., 2013; Kanekar and Aruin, 2014a,b; Pereira et al., 2014; Valles et al., 2014; Aruin et al., 2015; Claudino et al., 2017; McKee and Neale, 2019; Curuk et al., 2020; Yin et al., 2020; Molnar et al., 2021; Wang and van den Bogert, 2021; Zelei et al., 2021; Mohebbi et al., 2022), 1 study estimated the time delay in a stick balancing posture configuration (Nagy et al., 2020), 1 study used stepping, jump and squat postures for estimation (Murai et al., 2010), 1 study estimated the time delay during balancing a virtual inverted pendulum (Loram et al., 2009), while 2 other studies examined postures in supine position (Fujio et al., 2016), and when the subjects stood on their hands (Blenkinsop et al., 2016), respectively (Figure 6).

### 3.6 Types of perturbations

**FIGURE 7:**
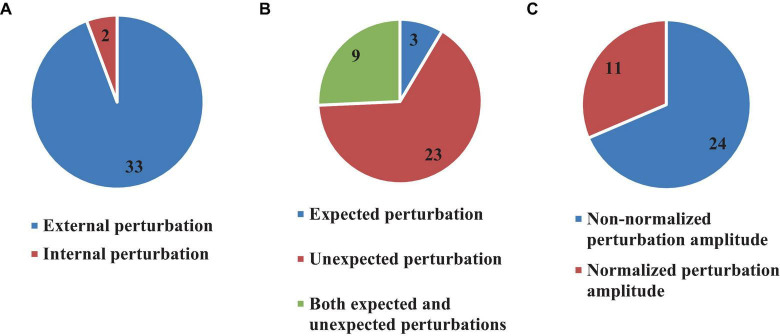
Number of articles which used external or internal **(A)**, expected and unexpected **(B)**, and Normalized or Non-normalized perturbation **(C)**.

Typical to balance investigations, most studies included in this review used induced perturbations (35 articles, 76%) (Radebold et al., 2000, 2001; Brown et al., 2001; Leinonen et al., 2001; Peterka, 2002; Redfern et al., 2002; Dimitrova et al., 2004; Granata et al., 2004; Peterka and Loughlin, 2004; Cholewicki et al., 2005; Reeves et al., 2005, 2009, 2018; Mochizuki et al., 2008; Welch and Ting, 2008, 2009; Loram et al., 2009; Santos et al., 2010; Borghuis et al., 2011; Mohapatra et al., 2012; Weaver et al., 2012; Liebetrau et al., 2013; van Drunen et al., 2013; Kanekar and Aruin, 2014a,b; Aruin et al., 2015; Blenkinsop et al., 2016; Fujio et al., 2016; Claudino et al., 2017; van Dieen et al., 2018; Curuk et al., 2020; Yin et al., 2020; Wang and van den Bogert, 2021; Zelei et al., 2021; Mohebbi et al., 2022) and estimated the time delay of the body response immediately after the perturbation. Two types of perturbations were used in the reviewed studies: internal and external (Figure 7).

In external perturbations, mechanical forces or initial conditions are typically used to destabilize the body (Figure 7). On the other hand, internal perturbations usually rely on a weak electrical current which triggers imbalance. Following this definition, 33 (71.7%) (Radebold et al., 2000, 2001; Brown et al., 2001; Leinonen et al., 2001; Peterka, 2002; Redfern et al., 2002; Dimitrova et al., 2004; Granata et al., 2004; Peterka and Loughlin, 2004; Cholewicki et al., 2005; Reeves et al., 2005, 2009, 2018; Mochizuki et al., 2008; Welch and Ting, 2008, 2009; Santos et al., 2010; Borghuis et al., 2011; Mohapatra et al., 2012; Weaver et al., 2012; Liebetrau et al., 2013; van Drunen et al., 2013; Kanekar and Aruin, 2014a,b; Aruin et al., 2015; Blenkinsop et al., 2016; Fujio et al., 2016; Claudino et al., 2017; van Dieen et al., 2018; Curuk et al., 2020; Yin et al., 2020; Wang and van den Bogert, 2021; Zelei et al., 2021) articles used externally based perturbations, while 2 articles (4.3%) (Loram et al., 2009; Mohebbi et al., 2022) used internal perturbations. Importantly, the perturbation was “expected” by the subjects in only 3 articles (8.5% out of 35 articles) (Brown et al., 2001; Kanekar and Aruin, 2014b; Curuk et al., 2020), while 23 (65.7% out of 35 articles) articles, used “unexpected” perturbation (Radebold et al., 2000, 2001; Peterka, 2002; Redfern et al., 2002; Dimitrova et al., 2004; Granata et al., 2004; Peterka and Loughlin, 2004; Cholewicki et al., 2005; Reeves et al., 2005, 2009; Welch and Ting, 2008, 2009; Loram et al., 2009; Borghuis et al., 2011; Liebetrau et al., 2013; van Drunen et al., 2013; Blenkinsop et al., 2016; Claudino et al., 2017; van Dieen et al., 2018; Yin et al., 2020; Wang and van den Bogert, 2021; Zelei et al., 2021; Mohebbi et al., 2022). Both “expected” and “unexpected” perturbations were included in the experimental protocols of 9 studies (25.8% out of 35 articles) (Leinonen et al., 2001; Mochizuki et al., 2008; Santos et al., 2010; Mohapatra et al., 2012; Weaver et al., 2012; Kanekar and Aruin, 2014a; Aruin et al., 2015; Fujio et al., 2016; Reeves et al., 2018) (Figure 7). Most of the reviewed articles did not consider the amplitude of the perturbation (24, 68.5%) (Brown et al., 2001; Leinonen et al., 2001; Peterka, 2002; Redfern et al., 2002; Dimitrova et al., 2004; Granata et al., 2004; Peterka and Loughlin, 2004; Mochizuki et al., 2008; Welch and Ting, 2008, 2009; Loram et al., 2009; Reeves et al., 2009, 2018; Borghuis et al., 2011; Mohapatra et al., 2012; Weaver et al., 2012; Liebetrau et al., 2013; van Drunen et al., 2013; Blenkinsop et al., 2016; Fujio et al., 2016; van Dieen et al., 2018; Yin et al., 2020; Wang and van den Bogert, 2021; Mohebbi et al., 2022), although 11 (31.5%) (Radebold et al., 2000, 2001; Cholewicki et al., 2005; Reeves et al., 2005; Santos et al., 2010; Kanekar and Aruin, 2014a,b; Aruin et al., 2015; Claudino et al., 2017; Curuk et al., 2020; Zelei et al., 2021) articles used normalized perturbation in their experimental tests (Figure 7).

### 3.7 Types of computational approaches for time delay estimation

**FIGURE 8:**
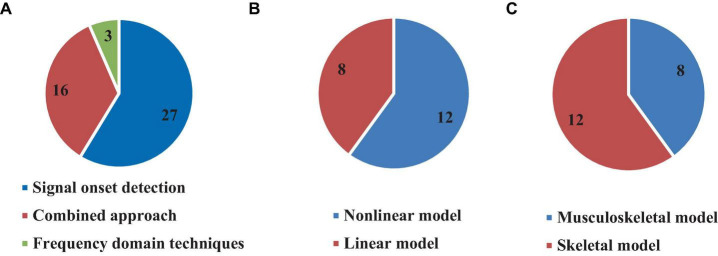
Number of articles based on using different approaches **(A)**, linear or non-linear model **(B)**, and modeling muscular system **(C)** for time delay estimation.

The reviewed studies used different computational approaches for estimating time delay, both in time and frequency domains. Direct experimental data analysis was used in 27 (58.7%) articles by detecting the signal onset (Radebold et al., 2000, 2001; Brown et al., 2001; Leinonen et al., 2001; Adkin et al., 2002; Redfern et al., 2002; Dimitrova et al., 2004; Granata et al., 2004; Cholewicki et al., 2005; Reeves et al., 2005, 2018; Mochizuki et al., 2008; Loram et al., 2009; Santos et al., 2010; Borghuis et al., 2011; Mohapatra et al., 2012; Weaver et al., 2012; Liebetrau et al., 2013; Kanekar and Aruin, 2014a,b; Pereira et al., 2014; Aruin et al., 2015; Blenkinsop et al., 2016; Fujio et al., 2016; Claudino et al., 2017; Curuk et al., 2020; Mohebbi et al., 2022), in which time delay was estimated by computing the time between the instant of perturbation and initial response in the form of kinetic and/or kinematic data. Most studies following this approach used EMG, where they computed the time delay between the perturbation instant and when the EMG signal increased more than twice the value of its standard deviation in a defined time window prior to the perturbation (see Table 3). Other studies estimated the time between the perturbation instant and when the COM or the COP reaches to the first or second peak, as well as when it is increased twice the value of its standard deviation in a defined previous time window. In general, the sensorimotor control system responds to a particular perturbation so that it can be identified by the COM or the COP. Some studies defined the first or second local maximum of these signal as the initial point of the body response to the perturbation. In fact, the sensorimotor control system responses to the perturbation so that it can be identified by COM or COP and some studies defined the first or second local maximum of those signal as the initial point of the body response to the perturbation (see Table 3). On the other hand, 16 articles (34.7%) used a combined time domain methodology consisting of simulation and optimization to estimate the time delay (Peterka, 2002; Maurer and Peterka, 2005; Masani et al., 2008; Vette et al., 2008; Welch and Ting, 2008, 2009; Reeves et al., 2009; Murai et al., 2010; Sovol et al., 2010; van Drunen et al., 2013; Valles et al., 2014; van Dieen et al., 2018; Yin et al., 2020; Molnar et al., 2021; Wang and van den Bogert, 2021; Zelei et al., 2021; Figure 8). For example, multiple studies developed a model, either musculoskeletal considering the muscles or without muscles, then used an optimization strategy to minimum the error between the produced kinematic trajectory or muscle activation by the model and experimental data in order to estimate the time delay and other defined parameters (e.g., joint stiffness). In addition, other studies used data fitting, regression models, neural networks, and Kalman filter approaches to match the simulated trajectories with experimental data (Peterka, 2002; Reeves et al., 2005; Murai et al., 2010; McKee and Neale, 2019).

Finally, some studies estimated the time delay in the frequency domain. In this approach, the data was transformed from the time domain to the time-frequency domain, and the time delay was estimated by measuring the time period between the perturbation instant and the first peak in the time-frequency domain (Peterka and Loughlin, 2004). One study (Nagy et al., 2020) used the cepstral approach (resulting from the inverse Fourier transform of a signal (Shokouhyan et al., 2023)) to estimate the time delay in stick balancing. Various weights and colors of sticks were employed to estimate two reaction times including both visual and tactile perception (Maurer and Peterka, 2005).

### 3.8 Linear and non-linear simulated models

Several studies included in this review used simulated models for time delay estimation (20, 43.4%) (Peterka, 2002; Peterka and Loughlin, 2004; Maurer and Peterka, 2005; Masani et al., 2008; Vette et al., 2008; Welch and Ting, 2008, 2009; Reeves et al., 2009; Murai et al., 2010; Sovol et al., 2010; Liebetrau et al., 2013; van Drunen et al., 2013; Valles et al., 2014; van Dieen et al., 2018; McKee and Neale, 2019; Nagy et al., 2020; Yin et al., 2020; Molnar et al., 2021; Wang and van den Bogert, 2021; Zelei et al., 2021), among which 15.2% used non-linear models (Maurer and Peterka, 2005; Murai et al., 2010; Liebetrau et al., 2013; van Drunen et al., 2013; McKee and Neale, 2019; Yin et al., 2020; Wang and van den Bogert, 2021) while 28.2% used linear models (Peterka, 2002; Peterka and Loughlin, 2004; Masani et al., 2008; Vette et al., 2008; Welch and Ting, 2008, 2009; Reeves et al., 2009; Sovol et al., 2010; Valles et al., 2014; van Dieen et al., 2018; Nagy et al., 2020; Molnar et al., 2021; Zelei et al., 2021; Figure 8). However, only 8 (40% of studies which used model) studies included the muscular system in their model to estimate the time delay and other parameters (Vette et al., 2008; Welch and Ting, 2008, 2009; Murai et al., 2010; Liebetrau et al., 2013; van Drunen et al., 2013; van Dieen et al., 2018; Yin et al., 2020), while 12 (60%) articles estimated the time delay without modeling the muscular system (Peterka, 2002; Peterka and Loughlin, 2004; Maurer and Peterka, 2005; Masani et al., 2008; Reeves et al., 2009; Sovol et al., 2010; Valles et al., 2014; McKee and Neale, 2019; Nagy et al., 2020; Molnar et al., 2021; Wang and van den Bogert, 2021; Zelei et al., 2021; Figure 8).

## 4 Discussion

The main goal of this PRISMA-guided scoping review was to summarize and integrate different theoretical, experimental, and meta-approaches used for the past couple of decades to estimate the time delay encountered by the human sensorimotor control system during stabilization tasks. The review of 46 articles reflected a major discrepancy in the range of the estimated time delays. The following discussion points aim to elucidate some reasons hypothesized behind such discrepancy.

### 4.1 The definition of time delays and impact of analytical approaches and experimental protocol

As previously mentioned, the time delay incurred during stabilization can be divided into four basic types: delay in sensory detection, transportation delay (back and forth), CNS processing delay, and electromechanical delay. The articles included in this review used various definitions for the time delay which they estimated. Most of the articles estimated the total time delay consisting of the sum of the 4 basic delays (above 150 ms, see subsection “3.3 Types of estimated time delay”). However, one article (Mochizuki et al., 2008) estimated the cortical time delay, which was lower than total time delay due to the elimination of CNS processing time. Another study estimated the neural transmission time delay (Murai et al., 2010) based on a neural network and optimization approach using EMG and kinematic data. Other studies [60–61] only assessed the muscle activation time delay in anticipatory postural adjustments (APA) conditions in healthy individuals and patients, in which the muscle activation time delay value was less than 150 ms in healthy individuals. Therefore, in an effort to compare studies, it is critical to understand the type of time delay used. Special attention needs to be paid to a study’s methodological approach and particular definition of the time delay. Different evaluation methodologies, data analyses and experimental protocols can result in different values, even in studies with the same time delay definition. While many studies reviewed here used EMG data to estimate the total time delay (see subsection ı0), other investigations used kinematic and COP data. Moreover, different approaches of analyses could cause the final estimated time delay value to vary. For example, some of the reviewed articles based their results on the time between a perturbation instant and the COM peak or the first peak of COP, while others measured the time between the perturbation instant and the time when the EMG signal is increased to more than of 2 or 3 times of the standard deviation of the signal before the perturbation. Since muscle activation does not represent the same instant as COP or COM reaches its peak, these different analyses approaches can lead to different estimated time delay values.

Finally, different experimental protocols can yield different values of estimated time delay. Most of the reviewed articles used perturbation-based experiments and/or simulations for time delay estimation. While some of these studies used normalized amplitudes of perturbation, it is critical to understand that the amplitude of perturbation can change the value of the time delay. Higher perturbation amplitudes can induce a reflex response. Thus, part of the time delay value will decrease due to the absence of CNS processing and signal transmission time delay (Kanekar and Aruin, 2014a,b; Aruin et al., 2015; Curuk et al., 2020). Furthermore, whether a perturbation is expected or not could also affect the value of the estimated time delay. In expected perturbations, the CNS could retrieve pre-information to predict the perturbation instant, which yields a more stable response and may reduce the time delay through the leverage of pre-information and pre-processing (Fujio et al., 2016; Curuk et al., 2020). On the other hand, pre-information, or pre-muscle activation, could also help the sensory motor control system to produce a better response against the perturbation. For example, a vocal cue (e.g., by the experimenter or device) before the perturbation could aid the participant to estimate the instant of perturbation. In studies which released a weight attached to the participants’ body to simulate the perturbation, the participants pre-activated some of their muscles before the release (i.e., perturbation) instant. This led the active muscles to enhance the body’s stability against the perturbation, and hence the sensory motor control system could stabilize the body in a less time. Such learning effect is also seen in repeated and practice trials (Peterka and Loughlin, 2004), where participants learn how to stabilize their bodies faster. Thus, the learning effect can reduce the estimated time delay as compared to trials conducted without prior training and those that lack multiple repetitions.

Eventually, the direction of perturbations (AP or ML) may also change the CNS processing time and lead to multiple estimated time delay values within the same experimental protocol. The stabilization strategy of the body is typically different in AP as compared with ML directions, which leads the CNS to use different strategies that may impact the value of the time delay. Moreover, the type of postural balance, mentioned above, is also important in estimating the time delay. Various postures can lead to different time delay values due to differences in the distance between the specific sensory systems/sensors and CNS, as well as the differences in actuated muscle groups. As previously mentioned, several strategies are used by the CNS to stabilize the body in different postures (e.g., stick balancing and balancing during hand stance, vs. simple standing stance).

### 4.2 Which model for time delay estimation?

Some of the articles reviewed here estimated the time delay based on experimental data, while others used simulation models with or without experiments (see subsection ı0). In general, if a particular study aims to estimate other parameters, in addition to time delay, developing a model is a good approach. Some of the reviewed articles used linear or non-linear models and various types of analyses, such as optimization, regression and ML, to minimize the error between the simulated kinematic and/or kinetic trajectories and the experimental data to estimate time delays, as well as other parameters such as joint stiffness. This approach requires the development of the best representative model toward optimizing both accuracy and precision. Developing faithful simulation models with sufficient complexity to align with physiology is very challenging for two main reasons. First, comprehensive non-linear models, must include non-linear dynamics, various uncertainties, non-linear sensory, control and data processing information, variant time delays, as well as muscle non-linear characteristics (Wang and van den Bogert, 2021). Such models require complicated optimization algorithms to match the experimental data. In contrast, simple linear models used for time delay estimation may yield good results within certain conditions and constraints.

For instance, Reeves et al. (2009) utilized a simple 2 DOF linearized model and estimated the time delay by optimizing the error between model and experimental data. The model worked except for movements outside the defined range, emphasizing that when using a linear model, caution should be exercised to restrict the results to the assumed range of motion. The second challenge encountered in modeling is related to estimating other parameters in addition to time delay. For instance, some of the reviewed studies (Peterka, 2002; Peterka and Loughlin, 2004; Maurer and Peterka, 2005; Masani et al., 2008; Mochizuki et al., 2008; Vette et al., 2008; Welch and Ting, 2008, 2009; Sovol et al., 2010; van Drunen et al., 2013; Valles et al., 2014; Blenkinsop et al., 2016; van Dieen et al., 2018; McKee and Neale, 2019; Yin et al., 2020; Wang and van den Bogert, 2021; Zelei et al., 2021; Mohebbi et al., 2022) used modeling to estimate ankle torque, joint damping, stiffness, as well as the time delay, adding to the level of their perspective model’s complexity. Other studies (Maurer and Peterka, 2005; Masani et al., 2008; Vette et al., 2008; Murai et al., 2010; Valles et al., 2014; Yin et al., 2020) attempted to estimate at least one type of time delay, individually, as part of total time delay. Based on the reviewed studies, the following is recommended: (1) Researchers should always start by defining the time delay that they plan to estimate. Subsequently, a well-designed clinical study can be conducted based on a standardized protocol which considers all key relevant perturbation characteristics, including direction, nature (internal or external), intention (expected or unexpected), normalization and pre-sensory information or pre-muscle activation; (2) Based on the literature, kinetic-based signals, such as EMG, may be more effective in identifying the post perturbation initial response as changes in their amplitude are more easily detected as compared with kinematic signals such as COP or COM; and (3) Using a model in addition to the clinical tests not only can increase the time delay accuracy, but may also be beneficial in estimating other passive elements, provided that the model is validated and verified by the experimental data.

## 5 Limitations

There are several limitations in this scoping review that are noteworthy. Only English language publications have been included in the search strategy. Thus, some studies may have been overlooked. Furthermore, a meta-analysis could not be adopted because of the methodological heterogeneity among the different studies. Moreover, the lack of standardization prevented the use of common quality assessment tools, such as COSMIN taxonomy (Mokkink et al., 2010). The quality scoring system was generous in some of the qualification questions. For instance, basic information regarding the analytical technique was assigned a score of 1, which may have improved the quality analysis outcome. Quantification questions were designed based on potential effective factors on the time delay value. However, we may have missed other factors which could affect the time delay that were not considered in this scoping review. Future work would benefit from improving both the qualitative and quantitative assessment techniques and criteria.

## 6 Conclusion

This scoping review was conducted to examine and integrate the different experimental, analytical, and computational approaches used in the past two decades to define and estimate the time delay embedded in the sensorimotor system in the context of biomechanical stability. The included reviewed articles demonstrated that the multiple definitions, experimental protocols, and analytical and computational approaches led to various estimated values of time delay. Some of the reasons behind this discrepancy are the lack of a standard definition, evaluation methodology, experimental protocol, and computational approaches. Moreover, the different studies reviewed here revealed diverse perturbation characteristics, including direction, nature (internal or external), intention (expected or unexpected), normalization and pre-sensory information or pre-muscle activation, all of which are crucial in estimating the time delay. Modeling with or without experimental validation can provide further insight, although caution should be exercised to balance fidelity of the model with complexity. Developing a model is particularly beneficial if the study aims to estimate other biomechanical parameters in association with the time delay. Future works should focus on clearly defining the nature of the studied delay and delineating appropriate experimental or theoretical approaches accordingly. Model validation is inevitable to ensure proper alignment with experimental data. Recent imaging and multimodal sensor fusion technologies and capabilities, in combination with AI and computational tools, provide an unprecedented opportunity to design sophisticated experiments and develop high fidelity models. This not only could help reveal the underlying mechanisms of the sensorimotor control system, but could also shed light on disease etiology and provide basis for the design of compensation/augmentation strategies for the aging and neurologically impaired populations.

## Data availability statement

The original contributions presented in the study are included in the article/supplementary material, further inquiries can be directed to the corresponding author.

## Author contributions

SS: Formal Analysis, Investigation, Methodology, Software, Writing – original draft. MB: Funding acquisition, Investigation, Methodology, Resources, Supervision, Validation, Visualization, Writing – review and editing. LW: Conceptualization, Methodology, Validation, Visualization, Writing – review and editing. FB: Funding acquisition, Methodology, Resources, Supervision, Validation, Visualization, Writing – review and editing. KK: Resources, Software, Validation, Visualization, Writing – review and editing.
